# Biological Properties of Bioceramic Sealers on Osteoblastic Cells: A Comparative Study

**DOI:** 10.1590/0103-6440202406037

**Published:** 2024-12-16

**Authors:** Angelita Piovezana Guerra, Danielle Gregorio, Gean Carlos Yamamoto, Nathalia Thalitha Bernardes dos Santos, Regina Celia Poli-Frederico, Luciana Prado Maia

**Affiliations:** 1 Graduate Program in Dentistry, University of North Parana (UNOPAR), Londrina, Paraná, Brazil; 2 Graduate Program in Rehabilitation Sciences, University of North Parana (UNOPAR), Londrina, Paraná, Brazil; 3 Graduate Program in Dentistry, University Anhanguera/Uniderp, Campo Grande, Mato Grosso do Sul, Brazil

**Keywords:** cytotoxicity, genotoxicity, biomineralization, endodontics, MTA

## Abstract

This study aimed to assess the biological properties of two ready-to-use bioceramic sealers (EndoSequence BC Sealer - EBCS; Bio-C Sealer - BCS) on osteoblastic lineage cells. MC3T3 osteoblast-like cells were exposed to extracts of bioceramic materials. Cytotoxicity was evaluated using the MTT method, genotoxicity was assessed by the micronucleus test and the expression of BMP1, BMP2 and ALP was measured by RT-qPCR, after 1, 3 and 7 days. Wound healing was monitored at 1, 2 and 3-day intervals using the scratch test. Statistical analysis involved a two-factor ANOVA, followed by the Tukey Test for multiple comparisons (α=0.05). Cytotoxicity assessment revealed no significant differences between the materials and the control group at any of the time points, indicating that neither material exhibited cytotoxic effects. However, both cements induced greater micronuclei formation compared to the control on days 1 and 7 (p<0.05) when evaluating genotoxicity. No significant differences between the groups were observed in wound healing at any of the time intervals. Both biomaterials upregulated the expression of BMP1, BMP2 and ALP. The two bioceramic sealers exhibited comparable biological properties, including cytocompatibility, promotion of wound healing, and upregulation of bone formation-related genes in osteoblast-like cells. While these results suggest the potential for safe clinical application, the observed genotoxicity warrants caution. Therefore, while the null hypothesis-that the two bioceramic sealers exhibit analogous properties-was accepted in terms of cytocompatibility and gene expression, further investigation is essential to fully ensure their safety and efficacy in bone-related procedures.

## Resumo

O objetivo deste estudo foi avaliar as propriedades biológicas de dois cimentos biocerâmicos (Bio-C Sealer -BCS; EndoSequence BC Sealer - EBCS) em linhagem osteoblástica. Células osteoblásticas MC3T3 foram expostas aos extratos dos materiais biocerâmicos. Após 1, 3 e 7 dias, a citotoxicidade foi avaliada pelo método MTT, a genotoxicidade pelo teste de micronúcleo e a expressão dos genes BMP1, BMP2 e ALP por RT-qPCR. A cicatrização da ferida foi avaliada nos tempos de 1, 2 e 3 dias pelo teste de arranhão. Os dados foram analisados por ANOVA 2 fatores, seguido pelo Teste de Tukey para comparações múltiplas (α=0,05). A avaliação da citotoxicidade não revelou diferenças significativas entre os materiais e o grupo controle em nenhum dos momentos, indicando que nenhum dos materiais apresentou efeitos citotóxicos. Entretanto, ambos os cimentos induziram maior formação de micronúcleos em relação ao controle nos dias 1 e 7 (p<0,05) na avaliação da genotoxicidade. Não houve diferença entre os grupos em nenhum dos tempos experimentais para a cicatrização de feridas. Ambos os biomateriais aumentaram a expressão de BMP1, BMP2 e ALP. Os dois cimentos biocerâmicos exibiram propriedades biológicas semelhantes, demonstrando citocompatibilidade, promoção da cicatrização de feridas e regulação positiva de genes relacionados à formação óssea em células osteoblásticas. Embora ambos exibem citocompatibilidade e regulação positiva de genes relacionados aos ossos, a genotoxicidade observada enfatiza a necessidade de consideração cautelosa em aplicações clínicas, exigindo mais investigações para garantir sua segurança e eficácia em procedimentos relacionados aos ossos.

## Introduction

In the field of endodontics, the introduction of bioceramic materials can be traced back to MTA (Mineral Trioxide Aggregate) ^1^. MTA-based materials have been widely studied and used due to their remarkable physicochemical and biological properties ^2^.

The first MTA-based obturation cement was formulated by Ângelus (Ângelus, Londrina, Brazil), and marketed under the name of “MTA Fillapex”. This product is characterized as a resin-based bioceramic cement, with a 13% bioactive load within its composition. MTA Fillapex is recognized for its biocompatibility, impressive radiopacity, and flow properties, which contribute to its good sealing capabilities and facilitate the healing of lesions ^3^. Nonetheless, one drawback associated with this material is its requirement for spatulation during preparation, which can potentially introduce errors.

Consequently, ready-to-use bioceramic cements were introduced as a convenient solution for endodontic obturation. Possessing a consistency conducive to effective canal sealing and exhibiting properties of significant relevance to the field of endodontics, these cements have been widely studied. Among the materials in this category, the EndoSequence BC Sealer (EBCS) (Brasseler USA, Savannah, GA) available in the North American market, while the Bio-C Sealer (Ângelus, Londrina, Brazil) is available in the Brazilian market.

The EBCS is a calcium silicate cement known for its high biocompatibility, non-toxicity, absence of aluminum content, antibacterial properties, and hydrophilicity. Its pH level is notably alkaline, and its setting reaction is initiated by the naturally occurring moisture within dentinal tubules. EBCS does not require mixing, ensuring a consistent and homogeneous product for all applications, thereby eliminating dosing errors ^4^. Unlike conventional cements, it does not undergo contraction during setting, its working time may be longer than 4 hours at room temperature and is conveniently available in a pre-loaded syringe format, with flexible tips for ease of application. It also exhibits excellent radiopacity, and the size of its nanoparticles enables rapid flow into the lateral and accessory canals, a capability made feasible through advancements in nanotechnology ^4^.

Since this product is not commercially available in Brazil, and the associated importation expenses render its clinical application economically impractical within the country, a Brazilian company has pioneered the development of a comparable alternative: the Bio-C Sealer (BCS). The BCS is characterized as a non-resinous bio ceramic cement, ready for use, boasting a substantial 65% bioceramic content within its composition. Its setting reaction is initiated by harnessing the naturally occurring moisture present in dentinal tubules ^5^. BCS exhibits notable features, including excellent radiopacity, the formation of hydroxyapatite upon hardening, hydrophilicity and chemical adherence to dentin. It is conveniently available in pre-loaded syringes, is biocompatible, possesses antibacterial properties, promotes accelerated healing, does not induce dental element discoloration, ensures effective drainage, and offers user-friendly application while preventing bacterial infiltration ^5^.

Given its recent introduction to the market, there are few studies evaluating the biological properties of BCS. In light of the progressively stringent regulations governing dental materials, it becomes imperative to conduct a series of biocompatibility and bioactivity tests. These tests serve the critical purpose of discerning the suitability of BCS for human application, ensuring it does not elicit adverse reactions or harm the patient's body. Moreover, they aim to substantiate the real effectiveness of BCS, building upon the established efficacy of its predecessor, MTA. Through comprehensive testing and evaluation, we can confidently determine its compatibility with human use and its ability to deliver outcomes consistent with its proven predecessor, MTA.

In view of the considerations outlined above, the primary objective of this study was to compare cytotoxicity, genotoxicity, wound healing kinetics, and the influence on expression of osteogenesis-related genes of Bio-C Sealer (BCS) with EndoSequence BC Sealer (EBCS) in an osteoblast-like cell line. The null hypothesis is that there is no difference between the two bioceramic sealers.

## Materials and Methods

### Culture of Osteoblastic cells

Mouse pre-osteoblastic MC3T3-E1 cells, subclone 14 (CRL-2594, ATCC, Manassas, VA, USA) was cultivated in alpha-minimum essential medium (Alfa-MEM; Gibco/Thermo Fisher Scientific, Waltham, MA, USA) supplemented with 10% fetal bovine serum (FBS; Gibco/Thermo Fisher Scientific) and 1% antibiotic and antimycotic (Gibco/Thermo Fisher Scientific), constituting the expansion medium. The cells were maintained in a humidified environment at 37^o^C with 5% CO_2_ and 95% atmospheric air. Upon reaching 80% confluence, the cells were subcultured and seeded into 96-well polystyrene plates (Corning Inc., Corning, NY, USA) at a concentration of 1x10^4^ cells per well. These cells were exposed to an osteogenic medium for 24 hours before being subjected to the extracts of the cements for MTT and micronucleus tests. The osteogenic medium consisted of the expansion medium supplemented with 7 mM β-glycerophosphate (Sigma-Aldrich, St. Louis, MO, USA) and 50 μg/mL ascorbic acid (Sigma-Aldrich).For the scratch test, the cells were subcultured in 12-well plates at a cell density of 1x10^5^ cells per well, while for RTq-PCR analysis, they were subcultured in 6-well plates at a concentration of 3x10^5^ cells per well, maintaining the same incubation conditions as described earlier.

### Manipulation of the Cements and exposure of the culture

The bioceramic endodontic cements (BCS and EBCS) were handled according to the manufacturer’s instructions to prepare cylindrical samples measuring 2x4 mm. This preparation was conducted under sterile conditions. Subsequently, immediately following manipulation, each sample was individually covered with sterile cotton wool that had been moistened with distilled water. These samples were then incubated for 48 h in a humidified environment at 37°C with 5% CO2 and 95% atmospheric air. There were no changes of the cotton cover during this incubation period. Following this incubation period, the osteogenic medium was exposed to experimental samples for 24 h, in the ratio of 2 samples/mL, according to ISO 10993-5:2009 ^(^ ^6^ standards. The extracts were filtered before being used in cell cultures. Osteogenic medium was used as negative control (C).

### Evaluation of cytotoxicity

Cytotoxicity was evaluated at days 1, 3 and 7 by the mitochondrial tetrazolium test (MTT; Sigma-Aldrich, St. Louis, MO, USA), according to ISO 10993-12:2012 ^7^. In brief, aliquots of MTT at 5 mg/ml in phosphate buffered saline solution (PBS; Gibco/Thermo Fisher Scientific) were prepared. Subsequently, the cells were incubated with a 10% MTT solution in an osteogenic medium for 4 hours at 37^o^C, in a humidified atmosphere containing 5% CO_2_. Following this incubation period, the entire solution was carefully aspirated, and 100 μL of dimethyl sulfoxide (DMSO) solution was added into each well under stirring for 5 minutes to ensure complete solubilization of the precipitate that had formed. The colorimetric measurement was carried out using a spectrophotometer (SpectratCount - Packard Instrument Company, USA), at a wavelength of 570 nm. The results were expressed as the relative percentage in comparison to the negative control, using the following formula: (DO_T_ - DO_B_) x (DO_C_ - DO_B_)) x 100, where DO = optical density; T = treatment; B = blank and C = control. Reduction in cell viability by more than 30% was considered cytotoxic ^6^.

### Genotoxicity Evaluation

Genotoxicity was assessed on days 1, 3 and 7, employing the micronucleus test (Mn). Briefly, the wells were subjected to three washes with PBS. Subsequently, the cells were fixed with 10% neutral formalin for 10 minutes, followed by another round of washing with PBS (Gibco/Thermo Fisher Scientific). After this preparatory phase, 200 μL of PBS and a drop of FluroShield with DAPI (Merck) were added into each well and the plates were stirred for 5 minutes under light protection. The examination of the wells was carried out using a fluorescence microscope (Eclipse Ti Nikon - UV-2ª filter, 330-380/420-700 nm wavelength of absorption/emission) by counting the number of micronuclei present within1000 cells/well with the assistance of the INFINITY ANALYZE Software program (Teledyne Lumenera, Canada).

### Wound Healing Evaluation (Scratch)

Wound healing analysis was conducted utilizing the scratch test ^8^ ^,^ ^9^. After 24 hours of cellular cultivation, a scratch was created at the bottom of each well using a 200 μL micropipette tip. Subsequently, the wells underwent three washes with PBS (Gibco/Thermo Fisher Scientific) to eliminate cellular debris. At this point, 1 mL of each of the extracts was added into the respective wells. The assessment of wound area was carried out on days 0, 1, 2 and 3. To measure the wound area, ImageJ (Java Image Processing and Analysis, National Institutes of Health, Bethesda, Maryland, USA) was utilized. The calculation of the percentage of wound area after 24, 48, and 72 hours was performed concerning the total area of the wound measured at 0 hours within the same well. The relative wound area (RWC) was determined through the following formula: RWC [%] = wound area [pixel] X 100 [%] / total area [pixel].

### Quantification expression of BMP1, BMP2 and alkaline phosphatase genes using qRT-PCR technique

For RNA extraction, the Cells-to-cDNA II kit (Reverse transcription without RNA isolation) (Gibco/Thermo Fisher Scientific) was used, according to the manufacturer's instructions. Subsequently, extracted RNA was quantified using a NanoDrop Lite spectrophotometer (Gibco/Thermo Fisher Scientific). The real-time PCR analysis was conducted using TaqMan Universal PCR Master Mix (Applied Biosystems, Waltham, Massachusetts, USA) and StepOnePlus System (Applied Biosystems) thermal cycler. The sequence of primers used in this study were: BMP1 (ENSG00000001684770), BMP2 (ENSG0000000125845) and alkaline phosphatase (ENSG0000000162551). The amplification reactions were performed within individual samples, each with a final volume of 20 μL and containing 1 μL of cDNA. These reactions adhered to the following conditions: an initial denaturation step at 95°C for 10 minutes, succeeded by 40 cycles comprising 15 seconds at 95°C and 1 minute at 60°C. For normalization, β-actin and GAPDH were employed as endogenous controls. To assess gene expression levels across the experimental groups, the comparative 2-ΔΔCt method was utilized. The results were presented as gene expression relative to the control group, with only QA (quantification cycle) values demonstrating a variation within ± 0.5 between individual sample reactions being considered valid.

### Data Statistical Analysis

Data were subjected to verification of normality and homoscedasticity by the Shapiro-Wilk test. Subsequently, two-factor analysis of variance (ANOVA) was used to compare the groups, followed by Tukey Test for multiple comparisons. A significance level of 5%. was utilized for all analyses. GraphPad Prism 7 software (GraphPad Software, California, USA) was employed for these statistical analyses.

## Results

### Cytotoxicity

**Figure 1:**
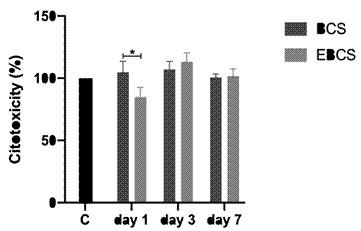
Cell viability of experimental groups throughout the experimental period. The bars indicate the mean ± standard deviation of the percentage of absorbance in relation to the control group. * = p<0.05

The results illustrated in Figure 1 indicate that, across all experimental time points, the materials did not exhibit statistically significant differences when compared to group C. However, on day 1 the EBCS group presented a significant reduction in cellular viability in comparison to BCS (p<0.05). As this reduction amounted to less than 30%, the material was not considered cytotoxic. No differences were observed in intra-group comparisons.

### Genotoxicity

**Figure 2:**
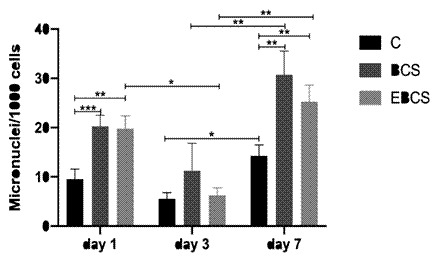
Number of changes consistent with genotoxicity in the different groups after 1, 3 and 7 days. The bars indicate mean ± standard deviation of the number of micronuclei/1000 cells. * = p<0.05

Across all groups, a decrease in the number of micronuclei from day 1 to day 3, was observed, followed by a subsequent increase at day 7 (Figure 2). This increase in micronuclei formation from day 3 to day 7 was statistically significant for all experimental groups (p<0.05). However, the reduction in micronuclei from day 1 to day 3 was statistically significant only for the EBCS group (p<0.05). In the intergroup analysis, both cements induced a higher formation of micronuclei compared to group C on days 1 and 7 (p<0.05). Notably, there was no statistically significant difference observed between the two cement groups.

### Wound healing (scratch)

**Figure 3:**
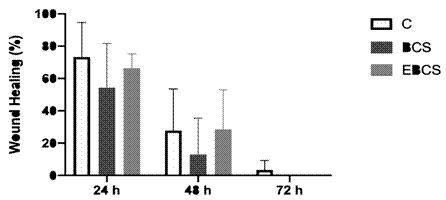
Wound area in the different experimental groups after 24, 48 and 72 h. The bars indicate the mean ± standard deviation of the percentage of the wound area in relation to the initial area.

In all experimental groups, a reduction in wound size was evident over time, yet there were no statistically significant differences observed among the groups at any of the experimental time points (Figure 3). Notably, only the BCS and EBCS groups demonstrated the capacity to fully close the wound area.

### PCR

In terms of gene expression, it was observed that BCS induced an increase in the relative expression of the ALP gene by a factor of 0.04 compared to the endogenous controls. Conversely, EBCS resulted in a more substantial increase, with a factor of 1.23. For the BMP1 gene, BCS led to an increase in relative expression by a factor of 0.29, while EBCS exhibited a greater impact with an increase of 1.31 times. Regarding the BMP2 gene, BCS induced an increase in relative expression by a factor of 0.30, while EBCS had a more pronounced effect, leading to an increase of 1.51 times.

## Discussion

The current study conducted a comprehensive evaluation of the biological properties of bioceramic endodontic materials, Bio-C Sealer and EndoSequence BC Sealer, in MC3T3 osteoblastic cell strain. Given the relative novelty of these products, it becomes imperative to conduct biocompatibility and bioactivity tests to ascertain their impact on biological systems. Such investigations are crucial for understanding the biological effects of these materials and ensuring their safe and effective utilization in dental applications.

In our study, the MTT test results indicated that the cements did not exhibit significant cytotoxicity. Importantly, there were no statistically significant differences observed in comparison to the control group across all experimental time periods. Our findings align with a study conducted by Zoufan *et al.* ^10^, which also reported similar results in EBCS cytotoxicity compared to Gutta Flow, AH Plus and Tubli-Seal in L929 fibroblast cells. They found that EBCS and Gutta Flow exhibited higher cytocompatibility when compared to AH Plus and Tubli-Seal. However, it is worth noting that Baraba *et al.* ^11^ reported different results in their evaluation of the cytotoxicity of EBCS and MTA Fillapex in L929 fibroblast cell line, with both cements exhibiting cytotoxicity.

Furthermore, López-García *et al.* ^9^ conducted research on the cytocompatibility and mineralization capacity of BCS, TotalFill BC Sealer and AH Plus in periodontal ligament stem cells (hPDLSCs). Their findings indicated that BCS and Total Fill BC Sealer demonstrated superior results in terms of cell viability, migration, cell morphology, and mineralization capacity, when compared to AH Plus. These findings were further supported by Kwak et al., ^12^ who demonstrated that BCS and ESBC exhibit lower cytotoxicity compared to AH Plus and TotalFill BC Sealer. Benetti et al. ^(^ ^13^ also reported similar results, indicating that BCS displayed greater cytocompatibility in comparison to MTA-Angelus and MTA-Fillapex. Notably, Kumar et al. ^14^ observed an initial cytotoxicity of BCS at 0 h, which significantly decreased over time, transitioning from severe cytotoxicity to noncytotoxic behavior.

Conversely, Okamura et al. ^15^ demonstrated that BCS resulted in a reduction in cell viability among V79 fibroblasts, isolated from hamster lung tissue. Similar findings were reported by Pedrosa et al., ^16^ who investigated the response of hPDLSCs to BCS, MTA Fillapex and Cimmo HP. Their research revealed that the pure extract of BCS, at the same dilution utilized in our study, exhibited cytotoxicity. Furthermore, Tolosa-Monfà et al. ^17^ reported a moderate (tending to slight) cytotoxicity of BCS in NIH 3T3 fibroblasts. These varying results highlight the importance of conducting comprehensive studies to assess the cytotoxicity and biocompatibility of dental materials in different cellular contexts and experimental conditions.

In addition to cytotoxicity, genotoxicity is an important property to evaluate because certain substances have the potential to induce cellular changes, including gene and chromosomal mutations ^18^. The results of the present study demonstrated a higher formation of micronuclei induced by the evaluated materials when compared to the control group. This suggests that both cements exhibit genotoxicity, with EBCS showing a somewhat higher propensity in this regard.

It's worth noting that the literature contains some variability in results regarding the genotoxicity of EBCS. For instance, Candeiro *et* al. ^19^ reported different results when comparing EBCS to AH plus in gingival fibroblasts using the micronucleus counting technique. In their study, EBCS exhibited a lower percentage of micronuclei formation when compared to AH Plus and control group. Similarly, Nair et al. ^20^ found evidence of lower genotoxicity in EBCS when compared to iRoot SP and eugenol zinc oxide in L929 fibroblasts, using the comet test. However, it's important to note that these studies primarily evaluated fibroblastic cells, and there were differences in the experimental conditions, including the dilution factor of the extracts used.

For BCS, to the best of our knowledge, this is the first study to assess *in vitro* genotoxicity. It's well-established that many chemicals can induce DNA damage in vitro, but the percentage of such damage is typically not significant *in vivo*, and therefore, it does not pose a risk to human DNA ^21^. In light of the positive results regarding genotoxicity in the present study, it underscores the necessity of conducting clinical studies to detect potential mutagenic effects and confirm the clinical implications of these findings ^22^. Clinical investigations will provide a clearer understanding of the real-world impact of these materials on patients.

In terms of the wound healing test, there were no statistically significant differences observed between the biomaterials and the control group regarding the rate of cellular migration at any of the experimental time points. However, it is worth noting that BCS displayed a tendency toward a smaller wound area at all time points, although this difference did not reach statistical significance. These findings align with a study by Sanz *et* al. ^23^ where the evaluation of BCS and EBCS HiFlow exhibited similar rates of cellular migration when compared to the control group. Additionally, in a study by López-García *et al.,* ^24^ EBCS and Ceraseal showed similar rates of cellular migration. However, EBCS demonstrated the highest migration rate in 72 hours when compared to the control group, while Endosal displayed a deceleration in cell migration throughout the analysis. Another study by Mestieri *et al.* ^25^ reported similar scratch test results in relation to EBCS. They observed that only EBCS displayed wound closure compared to AH Plus and MTA Fillapex. In our study, both BCS and EBCS demonstrated wound closure, which contrasts with the control group. However, further research and clinical studies are necessary to validate these observations and understand their clinical implications fully.

Considering the expression of BMP1, BMP2 and ALP genes, it is evident that both biomaterials induce an increase in the expression of all three genes. Notably, EBCS elicited a more substantial increase in gene expression compared to BCS. These findings align with a study conducted by Silva *et al.* ^(^ ^26^, which evaluated the biocompatibility of BCS, Sealer Plus BC and AH Plus in subcutaneous tissue of rats. They reported that BCS exhibited an increase in gene expression for osteocalcin, in contrast to Sealer Plus BC. AH Plus, on the other hand, did not express osteocalcin at any of the evaluated time points. Similarly, Rodríguez Lozano *et al.* ^8^ examined the expression of the ALP gene and found that the EBCS HiFlow and EBCS exhibited higher expression compared to the control group. However, AH Plus led to cell death, and the qPCR analysis could not be completed. For EBCS, the results of our study are consistent with Giacomino *et* al., ^27^ who assessed the gene expression of ALP using osteoblast precursor cells. They observed a significant increase in gene expression in EBCS and ProRoot ES when compared to Roth, AH Plus and the control group. These congruent findings across different studies emphasize the potential of BCS and EBCS to positively influence the expression of key genes related to osteogenesis and cellular activity, which is crucial for their effectiveness in dental applications and tissue healing.

This is the first study to evaluate genotoxicity, wound healing capacity, and the expression of BMP1, BMP2 and ALP genes induced by BCS in osteoblastic-like cells. The outcomes of our study contribute to the preliminary comprehension of this endodontic material, which holds significant relevance for clinical practice. It is imperative to acknowledge that findings derived from *in vitro* experiments cannot be directly extrapolated to the *in vivo* context, and, therefore, the results presented herein should be interpreted with caution. Nevertheless, the elucidation of these biological properties provides valuable insights into the potential clinical application of these endodontic cement.

Future studies focusing on mineralization potential and anti-inflammatory responses could further enhance our understanding of this material and offer valuable information for the development of improved therapeutic strategies. Furthermore, the results of this study should be complemented by further *in vivo* studies, encompassing genotoxicity assessments. By continuing to explore the biological characteristics and clinical performance of BCS and EBCS, researchers can contribute to the advancement of endodontic treatments and their overall effectiveness in dental practice.

## Conclusions

The results of this study demonstrated that the two bioceramic sealers exhibited comparable properties, leading to the acceptance of the null hypothesis. Both materials showed cytocompatibility, promoted wound healing, and induced upregulation of genes associated with bone formation in osteoblastic cells. However, the observation of some degree of genotoxicity underscores the need for further in-depth investigation to ensure the safety and efficacy of these materials in clinical applications.

## Data Availability

The data that support the findings of this study are openly available upon request.
